# Rapid visual *Candidatus* Liberibacter asiaticus detection (citrus greening disease) using simple alkaline heat DNA lysis followed by loop-mediated isothermal amplification coupled hydroxynaphthol blue (AL-LAMP-HNB) for potential local use

**DOI:** 10.1371/journal.pone.0276740

**Published:** 2022-10-25

**Authors:** Natkamol Thoraneenitiyan, Ilada Choopara, Suphachai Nuanualsuwan, Sirirat Kokpol, Naraporn Somboonna

**Affiliations:** 1 Program in Biotechnology, Faculty of Science, Chulalongkorn University, Bangkok, Thailand; 2 Department of Microbiology, Faculty of Science, Chulalongkorn University, Bangkok, Thailand; 3 Department of Veterinary Public Health, Faculty of Veterinary Science, Chulalongkorn University, Bangkok, Thailand; 4 Food Risk Hub Research Unit, Chulalongkorn University, Bangkok, Thailand; 5 Department of Chemistry, Faculty of Science, Chulalongkorn University, Bangkok, Thailand; 6 Microbiome Research Unit for Probiotics in Food and Cosmetics, Chulalongkorn University, Bangkok, Thailand; Huadong Research Institute for Medicine and Biotechniques, CHINA

## Abstract

An outbreak of citrus greening or Huanglongbing disease bacteria occurs in many areas. We sampled and identified an ongoing ~year 2020 orange tree endemic in northern Thailand as *Candidatus* Liberibacter asiaticus. We thereby developed a plant greening disease (*C*. Liberibacter asiaticus) detection assay using simple alkaline heat DNA lysis and loop-mediated isothermal amplification coupled hydroxynaphthol blue (AL-LAMP-HNB), and evaluated the developed assay for its feasibility as point-of-care detection on 65 plant leaf samples with 100–1×10^4^ copies of *C*. Liberibacter asiaticus or mocked injection compared with commercial DNA lysis kit and PCR-GE. Our assay is sensitive to 5–8.9 copies of *omp* (equaling 0.0056–0.01 fg) compatible with PCR-GE limit of detection. This ultra sensitive limit of detection could allow the disease detection before clinical apparent state of disease when *C*. Liberibacter asiaticus infection number is few, i.e. fewer than 100 copies of *C*. Liberibacter asiaticus. The assay is also specific with 6 degenerate primers targeting every strain of *C*. Liberibacter asiaticus *omp* from GenBank database, rapid (40 min total assay time), inexpensive (~2–3 USD/reaction), does not require sophisticated instrumentation, and has comparable assay accuracy (93.85–100% accuracy, 100% specificity, and 89.74–100% sensitivity) to bacterial DNA extraction by a commercial kit followed by PCR and gel electrophoresis (92.31% accuracy, 100% specificity, and 87.18% sensitivity) based on the real sample tests. Hence, the technique could be used in local or laboratory resource-restricted settings. The test result could be read by naked eyes through the color change from violet (negative) to sky blue (positive) for a *C*. Liberibacter asiaticus-infected specimen. Furthermore, this assay uses safe chemical reagents and, thus, is safe for the users.

## Introduction

A major economy and occupation in northern Thailand, such as Chiang Mai province, is orange tree (*Citrus* spp.) farming and exports. Farmers reported delayed detection of often endemic disease symptoms resulting in reduced size and quality of orange fruits, hence, massive widespread uses of chemical antibiotics (e.g. ampicillin, tetracycline and penicillin) were applied when the sign of this endemic disease was found. This endemic reduced the success of orange fruit harvests by ~20% from the year 2011 to 2014, and so on. Chanvatik et al. [1] reported >60% citrus greening diseases in orange orchards in Thailand in 2016. On the other hand, early detection of minimal number before clinical apparent disease and severe pathogen amount, if possible, might allow bioactive compound treatment to replace chemical antibiotics, and prevent unknown pathogen grafting (farmers and officers at Mae Fah Luang Foundation under Royal Patronage, personal communications) [1, 2]. Subsequently, the identification of this endemic orange tree outbreak in the studied region in northern Thailand and the development of a potential local-use assay for this disease detection at the early or non-symptomatic disease stage (a very high sensitivity assay) are critical.

Citrus greening or Huanglongbing disease is a worldwide destructive disease, provided that a non-culturable bacterial *Candidatus* Liberibacter asiaticus was specifically reported as the only disease pathogen in Thailand [3–5]. For conserved *C*. Liberibacter asiaticus detection, an outer membrane protein gene (*omp*) presents a common gene or protein target for the detection because *omp* has conserved parts of sequence intra-species [6]. Nonetheless, previous studies developed anti-Omp antibody detection [7, 8], while targeted nucleic acid-based amplification techniques like polymerase chain reaction (PCR) and loop-mediated isothermal amplification (LAMP) are accepted worldwide as gold standard laboratory diagnostics for the more sensitive and specific to a very low copy number of bacteria than the antibody detection [9, 10].

The PCR has been established for many disease pathogens for superior detection limit (<10 copy number) and specificity [9–11]. In conventional PCR detection, the procedures comprising the commercial DNA extraction kit followed by PCR for target nucleic acid region amplification and visualization of the result by agarose gel electrophoresis (GE) are widely accepted. However, these steps in the commercial DNA extraction kit and PCR-GE are laborious, expensive and inappropriate for local and restricted resource usage. The procedures also require a total of 4–4.5 h (0.5–1 h for DNA extraction, 3–3.5 h for PCR, and 0.5 h for GE).

Hence, after identifying the endemic pathogen cause in orange orchards in the studied region, we developed a plant crude DNA lysis following LAMP-colorimetric hydroxynapththol blue (AL-LAMP-HNB) assay that is specific and sensitive for the detection of *C*. Liberibacter asiaticus for potential local use to detect the disease at an early stage, enabling possible success with bioactive compound treatment. LAMP has been reported as an alternative to PCR for amplification and detection of specific nucleotide sequences [11–14]. LAMP uses *Bst* DNA polymerase and a set of 4–6 target-specific primers for autocycling and strand displacement DNA synthesis at a constant temperature of 60-65°C in 20–60 min. This method exhibits low price, convenience of use, high speed, and comparable sensitivity and specificity to the PCR [11, 13, 14]. During the LAMP reaction, Mg^2+^ is formed into insoluble magnesium pyrophosphate, and the concentration of Mg^2+^ in the solution can be titrated by a metal ion indicator such as hydroxynaphthol blue (HNB). LAMP-HNB, thereby, replaces the requirement of GE for detection of the LAMP product, and the addition of HNB since an initial stage of the LAMP allows a one-step reaction and simultaneous visualization of the result by a color change via naked eyes, from violet (negative) to sky blue (positive) [13, 15, 16]. Subsequently, we first successfully developed and tested an entire process of AL-LAMP-HNB from real samples to the colorimetric detection in a statistical number of *C*. Liberibacter asiaticus *omp* infected and non-infected *Citrus* leaf samples within 40 min.

## Materials and methods

### Field sample collections and identification of endemic orange tree pathogen

We surveyed and sampled using sterile scissors to cut leaves, buds, branches, and fruits with reported disease *C*. *reticulata* from 4 different farms in Chiang Mai province of Thailand, with permission from the area and farm owners, during midday on 3–4 December 2019. Farm owners reported this unknown endemic was a widespread problem in *Citrus* farms in Northern Thailand. All samples were collected and immediately transported on ice, for genetic extraction and identification of the disease pathogen. Total nucleic extraction was performed using GF-1 Plant DNA Extraction Kit (Vivantis Technologies, Selangor Darul Ehsan, Malaysia), measured quantity via A_260 nm_ spectrophotometry, and 16S rDNA PCR using universal primers 27F (5ˊ-AGAGTTTGATYMTGGCTCAG-3ˊ) and 1492R (5ˊ-TACCTTGTTACGACTT-3ˊ) [17, 18]. PCR (25 μL) comprised 12.5 μL EmeraldAmp® GT PCR Master Mix (TakaRa Bio, Shiga, Japan), 0.3 μM each primer, and 100 ng DNA (unless specified). The PCR conditions were 95°C for 4 min followed by 32 cycles of 94°C for 1 min, 55°C for 1 min, and 72°C for 2 min, with a final extension at 72°C for 10 min. Proper size (~ 1,466 base pairs (bp)) was determined by 1.5% agarose gel electrophoresis, and the full-length 16S rDNA PCR product was sequenced at Macrogen Inc. (Seoul, Korea). The sequence was determined species by BLASTN against the non-redundant GenBank database (E value < 0.001).

### Detection of *C*. Liberibacter asiaticus by commercial DNA extraction kit and PCR-GE

GF-1 Plant DNA Extraction Kit (Vivantis Technologies) was used to extract DNA from plant part samples following the manufacturer’s protocols. The PCR reaction for *C*. Liberibacter asiaticus *omp* (25 μL) comprised 12.5 μL EmeraldAmp® GT PCR Master Mix (TakaRa Bio, Shiga, Japan), 0.3 μM primers Cla-F3 and Cla-B3 (Table 1) (Cla-F and Cla-R were used to make full-length *omp* for copy number template), and 100 ng DNA (unless specified). Thermocycling conditions were as follows: 94°C for 4 min, followed by 35 cycles of 94°C for 1 min, 57°C for 1 min, and 72°C for 2 min, with a final extension at 72°C for 10 min. The PCR product was analyzed by 1.75% agarose gel electrophoresis (1,040 bp).

**Table 1:** Primer sequences of LAMP-HNB and PCR for *C*. Liberibacter asiaticus.

Primer	Sequence 5′-3′
Cla-F3	GAACAGA(A/T)GC(T/A)GCGACAG
Cla-B3	(C/T)GGGTTTAGAGTAGT(G/A)A(C/T)ATC
Cla-FIP	TTAACTCCAATGTA(A/G)GGAGTGAACA-ATGCCTCTATTGA(C/T)TACCAT
Cla-BIP	GGTCT(A/C)GAG(T/C)AAGTTTTGATGCCG–CAAGAT(T/A)GCTT(C/T)AGCCAATT
Cla-LF	CATATT(T/C)A(AAT(C/T)CGTATAGCTCAGCC
Cla-LB	AT(C/T)CGTATAGCTCAGCC
Cla-F	TAGTTCGCTCTCGATCTGCG
Cla-R	TCCAAACCCTGCCGCTAAAC

### Detection of *C*. Liberibacter asiaticus by simple alkaline heat DNA lysis and LAMP-HNB (AL-LAMP-HNB)

Simple alkaline heat DNA lysis was performed by cutting and grinding 200 mg of plant part sample into eight volumes of 0.2 M NaOH buffer. They were kept at room temperature for ~3 min and 5 μL was mixed with 45 μL 0.2 M NaOH buffer. The mixture was boiled at 75°C for 20 min and the tube was placed on ice to obtain the clear liquid at the top as the template for LAMP-HNB [19, 20]. For *C*. Liberibacter asiaticus *omp* LAMP, the degenerate primers were designed based on the multiple sequence alignments of all *C*. Liberibacter asiaticus *omp* sequences downloaded from GenBank, using Primer Explorer V5 program (http://primerexplorer.jp/lampv5e/index.html) and manual design (Table 1). Two inner-loop forward (Cla-LF) and backward (Cla-LB) primers were included to speed up the reaction to 20 min. The specificity of each primer was pre-screened by BLASTN against the GenBank database. The LAMP-HNB reaction mixture (15 μL) comprised 0.2 μmol l^-1^ primers Cla-F3 and Cla-B3, 1.6 μmol l^-1^ primers Cla-FIP and Cla-BIP, 1.4 μmol l^-1^ primers Cla-LF and Cla-LB, 1.4 mmol l^-1^ dNTP (SibEnzyme Ltd., Novosibirsk, Russia), 0.5 mol l^-1^ betaine (Sigma–Aldrich, St. Louis, Missouri, USA), 6 mmol l^-1^ MgSO_4_, 6 U of *Bst* DNA polymerase (large fragment; New England Biolabs, Beverly, Massachusetts, USA) along with 1× of thermoPol^TM^ Reaction Buffer (New England Biolabs), 120 μmol l^-1^ HNB, and the DNA template. The reaction was incubated at 60°C for 20 min, using a heat block incubator or a water bath. The result could be interpreted by naked eyes via violet (negative) or sky blue (positive) color change and was confirmed by 1.5% agarose gel electrophoresis.

### Study design to compare assay performance between conventional method (commercial DNA extraction kit and PCR-GE) and our developed method (crude DNA lysis and LAMP-HNB or LAMP-GE) using spiked-in *C*. Liberibacter asiaticus *omp*

To determine the performances of the conventional and our developed methods, statistically required sample numbers (N) were calculated according to the equation: N = (p (1-p) z^2^)/e^2^, given p at an average incidence of 60% [1], z score of 1.65 for 90% confidence interval, and e of 10% for margin of error. This yielded an N of 65. Therefore, we autoclaved 65 *C*. *reticulata* leaf samples and spiked-in *C*. Liberibacter asiaticus *omp* for 39 samples (equal to 60% infection incidence) (samples were equally and randomly injected through leaf veins with 100, 500, 1×10^3^, or 1×10^4^ copy numbers of *omp*) and sterile water for 26 samples. For the conventional and our developed methods, the protocols were as described above. The assay efficacy was calculated based on the following equations: sensitivity = true positive ÷ (true positive + false negative), specificity = true negative ÷ (true negative + false positive), false positive = 1 − specificity, false negative = 1 − sensitivity, and accuracy = (true positive + true negative) ÷ total samples.

## Results and discussion

Identification of the endemic disease pathogen in four *Citrus* orchards in Chiang Mai province of Thailand was successfully analyzed in leaves, buds, branches, and fruits. DNAs from each part were extracted from buds and leaves containing the relatively highest DNA amount per gram weight (S1A Table), and this DNA amount inferred total DNA (from the plant and microorganisms infecting plant DNAs). These DNAs were then analyzed for the pathogen cause of the disease by universal bacteria full-length 16S rRNA gene PCR (S1 Fig) and sequencing. The sequencing results were all identified to be *C*. Liberibacter asiaticus by BLASTN against the GenBank database (S1B Table).

**Fig 1:**
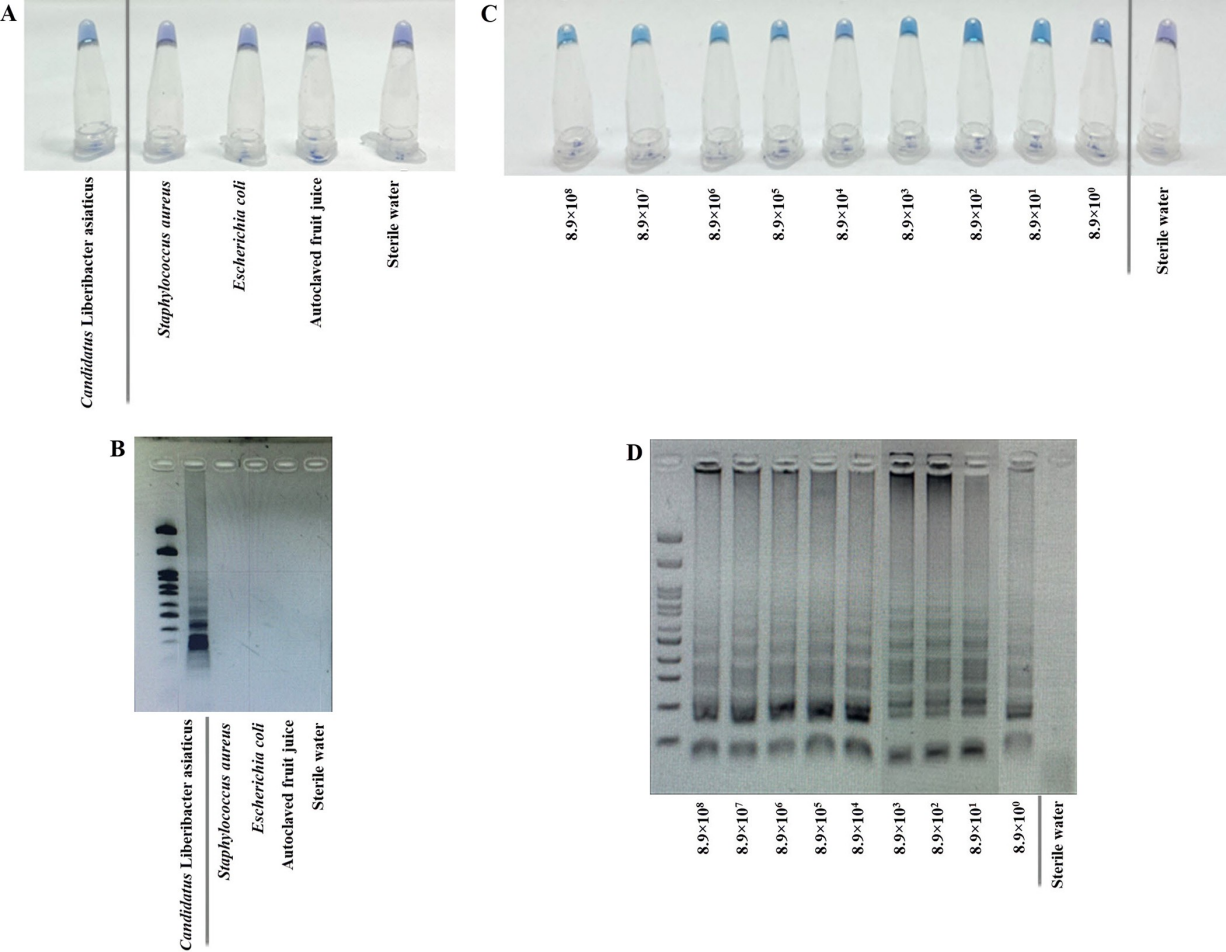
Specificity and sensitivity determination of our developed methods by LAMP-HNB (A and C) and LAMP-GE (B and D). Specificity assay (**A** and **B**) included positive (*C*. Liberibacter asiaticus) and negative (*Staphylococcus aureus*, *Escherichia coli*, autoclaved (*Citrus*) fruit juice, and sterile water) controls. Sensitivity assay (**C** and **D**) included 8.9×10^8^ to 8.9×10° copy numbers (equaling 1.0 ng to 0.01 fg) of *omp* from *C*. Liberibacter asiaticus and sterile water. In **B** and **D**, the first left lane was OneMARK 100 DNA ladder (Bio-Helix), and a positive LAMP reaction showed characteristic multiple ladder-like bands due to intercalating multiple loops of the LAMP product. Further limits of detection of LAMP-HNB, LAMP-GE, and PCR-GE assays at 5 and 1 copy numbers of *omp* are in S3 Fig.

**Fig 2:**
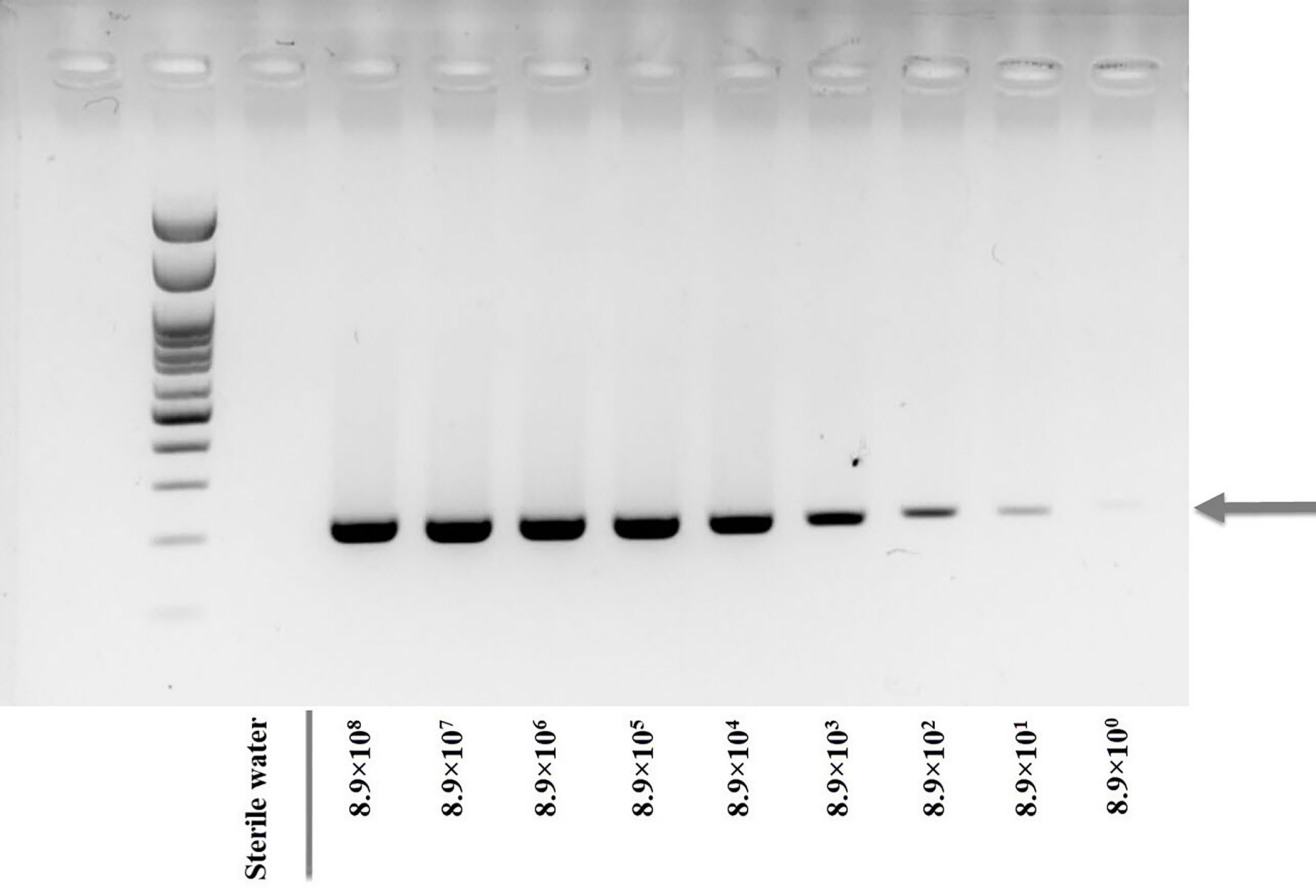
Sensitivity determination of PCR-GE using primers Cla-F3 and Cla-B3. The first left lane was OneMARK 100 DNA ladder (Bio-Helix). The sensitivity assay included sterile water (negative control) and 8.9×10^8^ to 8.9×10° copy numbers (1.0 ng to 0.01 fg) of *omp* from *C*. Liberibacter asiaticus.

In the following, we developed a LAMP-HNB assay for *C*. Liberibacter asiaticus detection based on *omp*, which is a standard gene for conserved *C*. Liberibacter asiaticus detection among molecular laboratories [6–8]. The *omp* sequences from all available *C*. Liberibacter asiaticus *omp* sequences from the GenBank database were aligned using ClustalW (http://www.megasoftware.net/), and degenerate primers (Table 1) were designed targeting six independent conserved regions of the aligned *omp* sequences. Each LAMP primer sequence specificity was also checked by BLASTN against the GenBank database. Different incubation temperatures (60-65°C) and times (20–60 min) were tested and the optimal LAMP-HNB incubation condition was at 60°C for 20 min, using a simple heat block or water bath. This optimal incubation condition demonstrated proper specificity on positive and negative controls (Fig 1A, i.e. *Staphylococcus aureus* was sometimes found on orange) [21], and the limit of detection as low as 8.9 copies of *C*. Liberibacter asiaticus using 10-fold serial diluents containing 8.9×10^8^ to 8.9×10° copy numbers (equivalent to 1.0 ng to 0.01 fg) of *C*. Liberibacter asiaticus *omp* (Fig 1C). For additional specificity assay, other negatives for our LAMP-HNB and LAMP-GE included *Staphylococcus epidermidis* and *Bacillus subtilis* (both species were generally found in orange whole trees and leaves) [21] (S2 Fig). To confirm the positive (sky blue) and negative (violet) color readouts by naked eyes, these LAMP-HNB products in Fig 1A and 1C were analyzed by GE, and a positive LAMP reaction showed characteristic ladder-like bands due to several target-specific primers intercalating to create multiple loops of the LAMP product (Fig 1B and 1D) [11–13]. Moreover, the 5 and 1 copy diluents were made and the detection limit, which remained at 8.9 copies for LAMP-HNB (S3A Fig), was found at 5 copies (0.0056 fg) for the LAMP-GE with ~50% reproducible results (S3B Fig: 2 of 4 lanes of 5 copies LAMP-HNB reactions showed LAMP products).

This limit of detection was compared to *omp* PCR-GE. The 10-fold serial diluents containing 8.9×10^8^ to 8.9×10° copy numbers (equivalent to 1.0 ng to 0.01 fg) of *C*. Liberibacter asiaticus *omp* were determined (Fig 2). Then, the lower copy (5 and 1 copies) tests were attempted and the PCR-GE was found able to detect as low as 5 copies (S3C Fig). This 5–8.9 copy limit of detection by the LAMP-HNB and LAMP-GE was, thereby, comparable with the conventional PCR-GE method, and this < 10 copy limit of detection by LAMP was indeed reported previously [11, 14, 16].

Moreover, the limit of detection of our developed LAMP assay at 5–8.9 copies was sufficient to determine the suspect Huanglongbing disease in *Citrus* spp. Stover and McCollum [22] reported the visual Huanglongbing disease appearance correlating with the *C*. Liberibacter asiaticus copy number for the whole tree and leaf samples, and used 16S rDNA real-time PCR to rate the severity appearance: ≥ ~100 copies (real-time PCR, Ct value ≤ ~32.5) were rated for visual Huanglongbing disease appearance; > ~5–99 copies (32.5 < Ct value < ~37.15) were for suspect or undetermined Huanglongbing disease appearance; and ≤ ~2.5 copies (Ct value ≥ ~39.5) were for no Huanglongbing disease appearance [23, 24]. Subsequently, our assay supports early disease detection allowing *Citrus* management before clinical severity appearance.

For potential local use, we additionally modified a simple heat alkaline DNA lysis [19, 20] and was firstly successful in using it together with LAMP-HNB (AL-LAMP-HNB) for real sample diagnostics of *C*. Liberibacter asiaticus from different plant parts in 40 min total time, requiring only a heat block or water bath. The crude DNA lysis step success in the combined AL-LAMP-HNB assay using raw plant samples could be explained by the low inhibitor problem by *Bst* DNA polymerase in LAMP, unlike *Taq* DNA polymerase in PCR [11, 25, 26]. The low inhibitor problem in LAMP avoids more expensive and laborious commercial DNA extraction kit steps, and helps minimize the cost and time of the AL-LAMP-HNB assay. Total assay time is only 40 min (20 min plant crude DNA lysis and 20 min LAMP-HNB) compared to 4–4.5 h total time required by conventional methods (0.5–1 h for DNA extraction by commercial kit, 3–3.5 h for PCR, and 0.5 h for GE), and the price is low at ~2–3 USD/reaction [27]. Furthermore, we evaluated the feasibility and compared the assay performance on a statistical number of real *Citrus* leaf samples (N = 65 samples) with vein-injected sterile water or *C*. Liberibacter asiaticus *omp* at 100–10,000 copies per reaction. Our developed AL-LAMP-HNB and AL-LAMP-GE assays showed the greater assay sensitivity (89.74–100%) and accuracy (93.85–100%) than the commercial DNA extraction kit and PCR-GE (sensitivity 87.18%, accuracy 92.31%) (Table 2). The zero to lower false negative rate in the AL-LAMP-HNB and AL-LAMP-GE assays is important because this limits the false detection when the plants indeed carried the infection [15]. The zero false positive rate in the AL-LAMP-HNB and AL-LAMP-GE assays is consistent with the fact that the LAMP-HNB reaction begins as negative color readout, and the reaction will turn to positive color readout only when the DNA has *C*. Liberibacter asiaticus (20 min is the minimal time that the positive color readout appears visible for the starting DNA template at 8.9 copies of *C*. Liberibacter asiaticus *omp*).

**Table 2:** Comparison of our developed assay (AL-LAMP-HNB and AL-LAMP-GE) to molecular conventional methods (commercial DNA extraction kit and PCR-GE) on 65 orange leaf samples (39 *C*. Liberibacter asiaticus-infected and 26 mock-infected leaves).

Test Statistics	Conventional Method (PCR-GE)	Our Developed Method	
		AL-LAMP-HNB	AL-LAMP-GE
No. of positive samples (39)	34 (TP^a^ = 34)	35 (TP = 35)	39 (TP = 39)
No. of negative samples (26)	31 (TN^a^ = 26)	30 (TN = 26)	26 (TN = 26)
Specificity (%)	100	100	100
Sensitivity (%)	87.18	89.74	100
False Positive (%)	0	0	0
False Negative (%)	12.82	10.26	0
Accuracy (%)	92.31	93.85	100
Undetermined^b^^a2^	0	0	0

^a^In parentheses, TP and TN represent true positive and true negative data of PCR-GE, AL-LAMP-HNB, and AL-LAMP-GE, accordingly.

^b^Undetermined samples in our developed assay refer to the number of nebulous-color LAMP-HNB results between blue (positive) and violet (negative). Here, there was no undetermined sample from real leaf sample assay for the conventional method, AL-LAMP-HNB, and AL-LAMP-GE.

**Fig 3:**
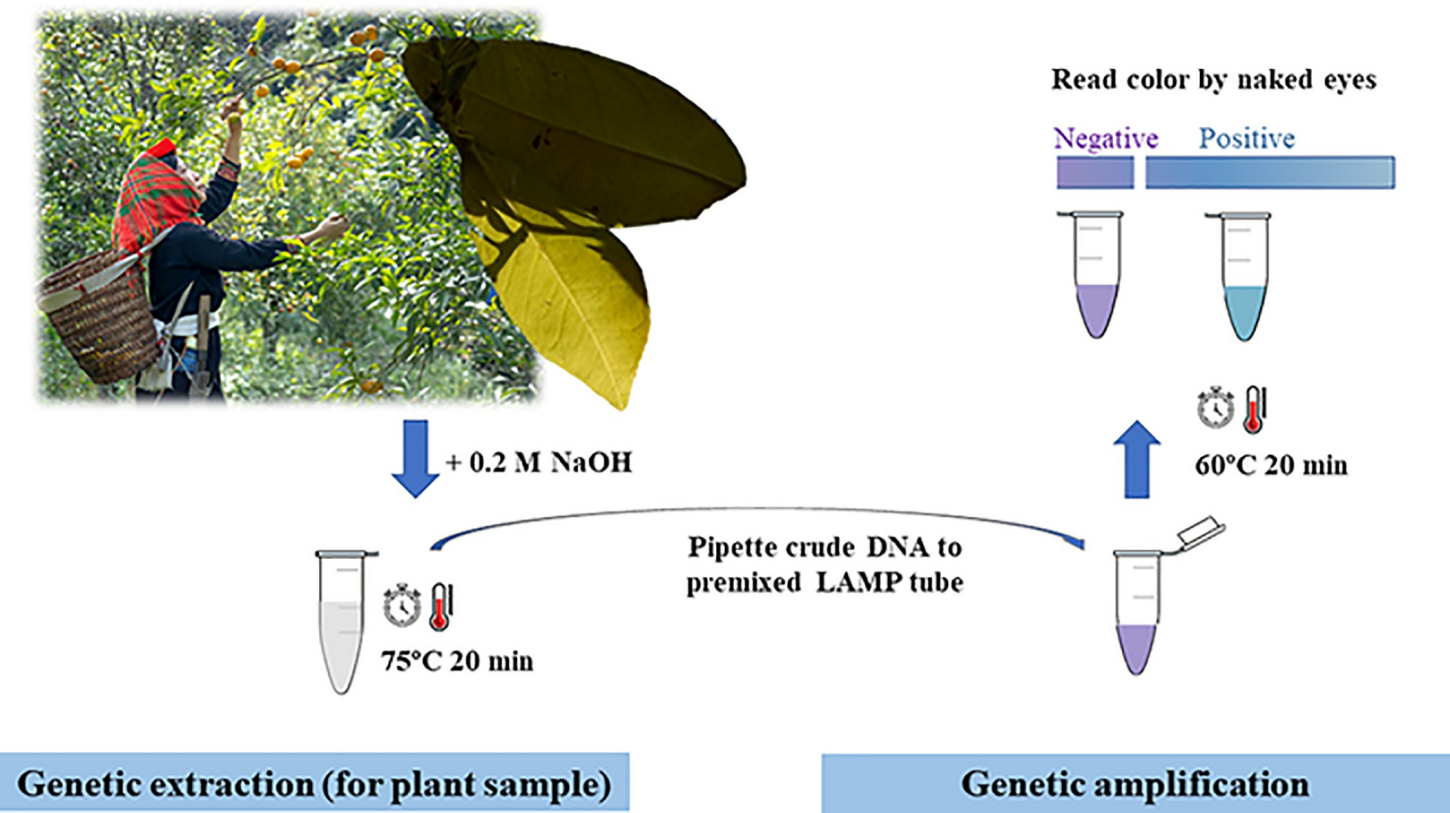
Graphical abstract of *C*. Liberibacter asiaticus AL-LAMP-HNB assay for potential local plant greening disease detection applications.

Following Rigano et al. [28] and Choi et al. [29] who stressed the importance of early and accurate *C*. Liberibacter asiaticus detection in plants and, hence, developed LAMP assays for this pathogen detection, our methods (Fig 3) further included a simple alkaline heat DNA lysis method for easy plant (or vector insect) sample crude DNA lysis in local settings, designed degenerated primers to target every strain of *C*. Liberibacter asiaticus, optimized the LAMP reaction condition to reduce the reaction time from 40–60 min to 20 min, and utilized a visible dye HNB as a one-step result readout to replace lateral flow dipstick (which requires an additional cost for biotin-labeled sequence specific dipstick preparation and a result readout requires a hybridization step of approximately 10 min) or the fluorescent dye (i.e. SYBR Green I (Invitrogen, New York, USA), which costs a much higher price compared with HNB dye). Moreover, our study evaluated an entire process of our developed methods (AL-LAMP-HNB and AL-LAMP-GE) on real plant samples with none or 100–1×10^4^ copies of *C*. Liberibacter asiaticus *omp*, in compared with the conventional method, and reported the comparable assay efficacy.

## Conclusions

Our developed AL-LAMP-HNB assay for *C*. Liberibacter asiaticus detection (Thai petty patent no. 2203002284) is simple, rapid, inexpensive, specific, sensitive, and does not require any complicated instrumentation. Evaluation of the assay over positive-negative controls and 65 infected real *Citrus* leaves (100–1×10^4^ copies per reaction) and non-infected samples showed the relative assay effectiveness compared to the conventional method by a commercial kit for DNA extraction and purification coupled with PCR-GE. The LAMP-HNB results can be read by direct visual inspection of the blue (positive) or violet (negative) colors, and the detection limit by naked eyes is as low as 8.9 copies (0.01 fg) of the target *omp*, or 5 copies (0.0056 fg) by LAMP-GE. This developed assay is, thereby, a promising tool for screening plant samples for *C*. Liberibacter asiaticus infection since early infection (very high sensitivity or asymptomatic) period in low resource settings. This could allow local plant greening disease detection before clinical apparent state of disease (when *C*. Liberibacter asiaticus infection number is fewer than 100 cells), with 93.85–100% diagnostic accuracy and cost-effective.

## Supporting information

S1 Fig Examples of 16S rDNA PCR electrophoresis gel of extracted DNA from orange leaf, bud, branch, and fruit samples (lanes 2–5), and lane 6 was sterile water. Lane 1 is OneMARK 100 DNA ladder (Bio-Helix, New Taipei City, Taiwan).
(TIF)

S2 Fig Specificity determination of our developed methods by LAMP-HNB (A) and LAMP-GE (B). Specificity assay (**A** and **B**) included positive (*C*. Liberibacter asiaticus) and negative (sterile water, *Staphylococcus aureus*, *Escherichia coli*, autoclaved (*Citrus*) fruit juice, *Staphylococcus epidermidis* and *Bacillus subtilis*) controls. In **B**, the first left lane was OneMARK 100 DNA ladder (Bio-Helix).
(TIF)

S3 Fig Limit of detection determination at 5 and 1 copy numbers of *C*. Liberibacter asiaticus *omp* and sterile water (negative control) by LAMP-HNB (A), LAMP-GE (B), and PCR-GE (C) assays. In (**B**)and (**C**), in addition to replicate tests of each assay in 5 and 1 copies templates, the left and right referred to two GE repeats.
(TIF)

S1 Table Average extracted DNA (ng) per gram from *C*. *reticulata* leaf, bud, branch, and fruit samples (A), and BLASTN of 16S rDNA query sequence against non-redundant GenBank database for identification of bacterial pathogen (B).
(PDF)

S1 File Original uncropped and unadjusted gel electrophoresis images of Fig 1D and S1 Fig. The other figures are of original images.
(PDF)

S1 Graphical abstract (TIF)
