# Supplementary figures and images for: Rapid visual Candidatus Liberibacter asiaticus detection (citrus greening disease) using simple alkaline heat DNA lysis followed by loop-mediated isothermal amplification coupled hydroxynaphthol blue (AL-LAMP-HNB) for potential local use
Source: PLoS One. 2022 Oct 25;17(10):e0276740. doi: 10.1371/journal.pone.0276740 (PMC9595546; doi:10.1371/journal.pone.0276740)

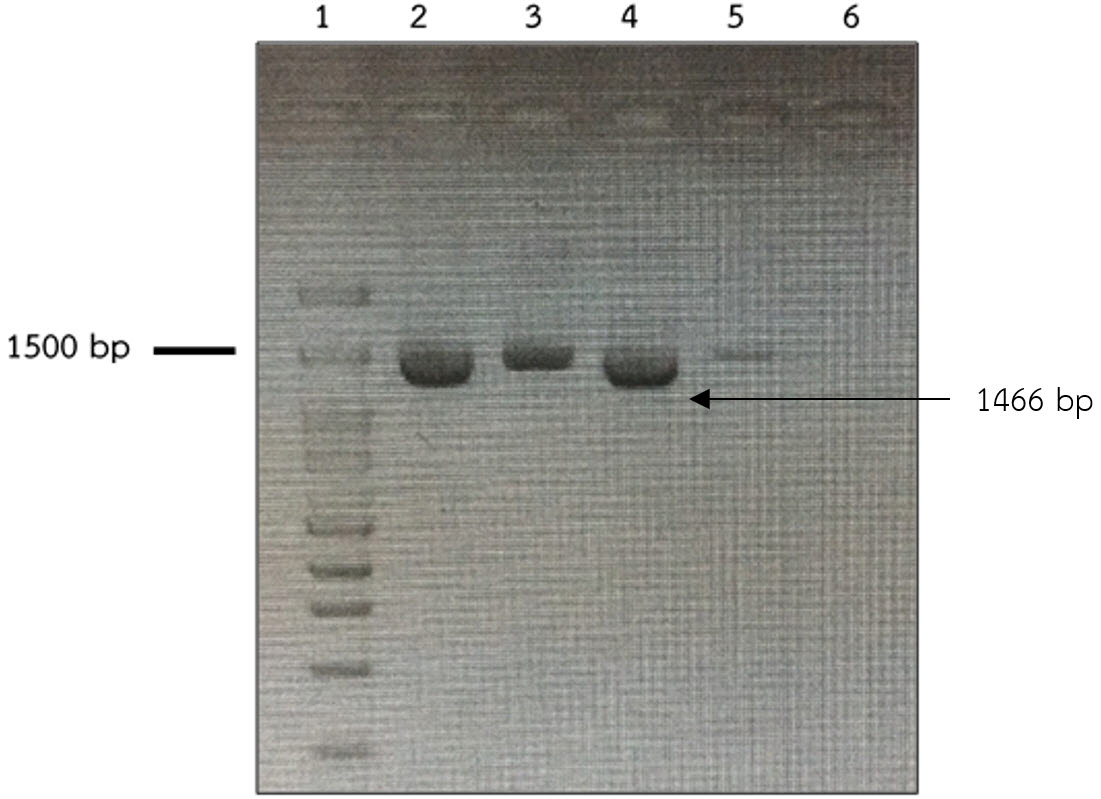

Supplement: S1 Fig — Lane 1 is OneMARK 100 DNA ladder (Bio-Helix, New Taipei City, Taiwan). (TIF) [file pone.0276740.s001.tif]

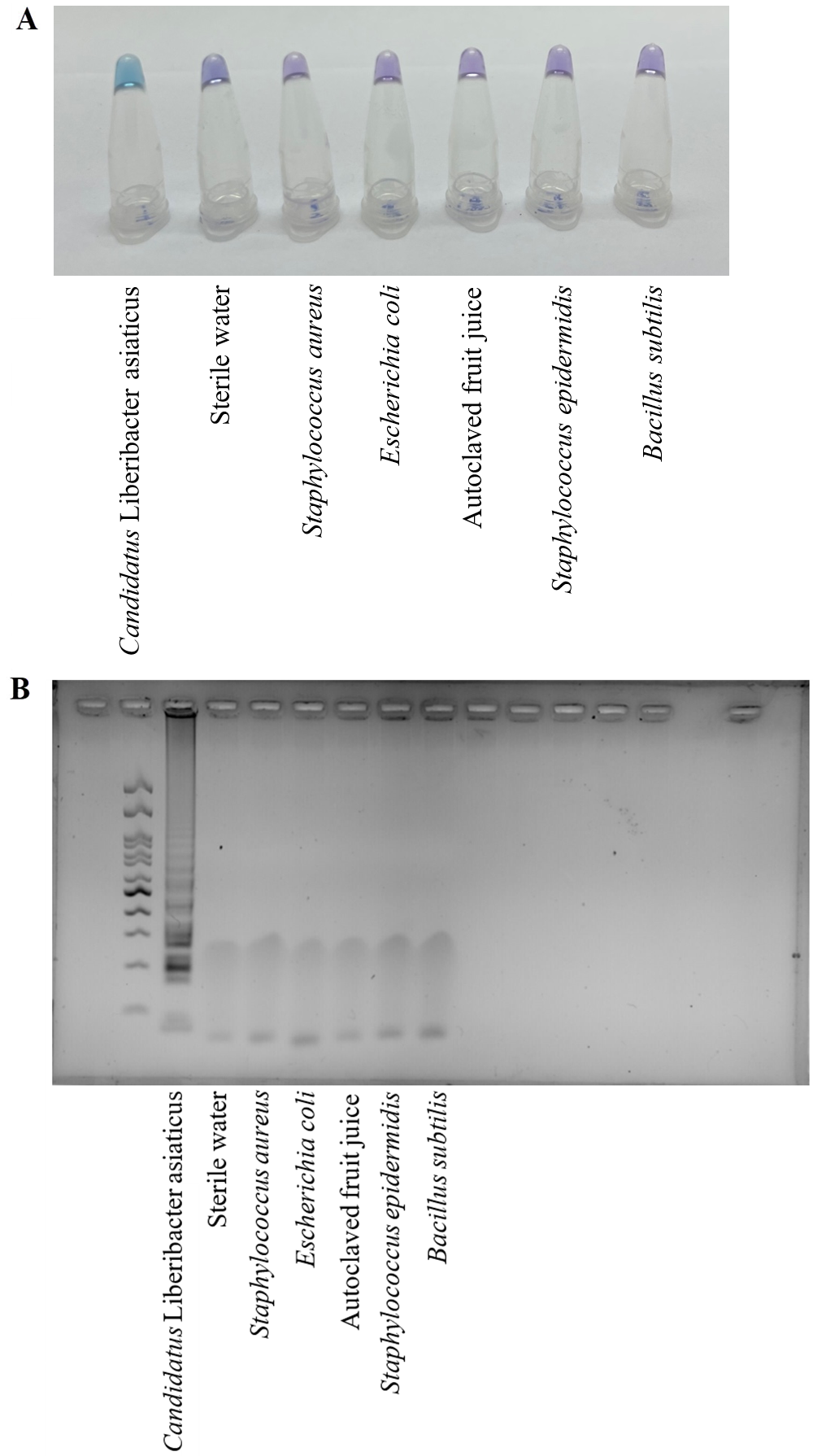

Supplement: S2 Fig — Specificity determination of our developed methods by LAMP-HNB (A) and LAMP-GE (B). Specificity assay (A and B) included positive (C. Liberibacter asiaticus) and negative (sterile water, Staphylococcus aureus, Escherichia coli, autoclaved (Citrus) fruit juice, Staphylococcus epidermidis and Bacillus subtilis) controls. In B, the first left lane was OneMARK 100 DNA ladder (Bio-Helix). (TIF) [file pone.0276740.s002.tif]

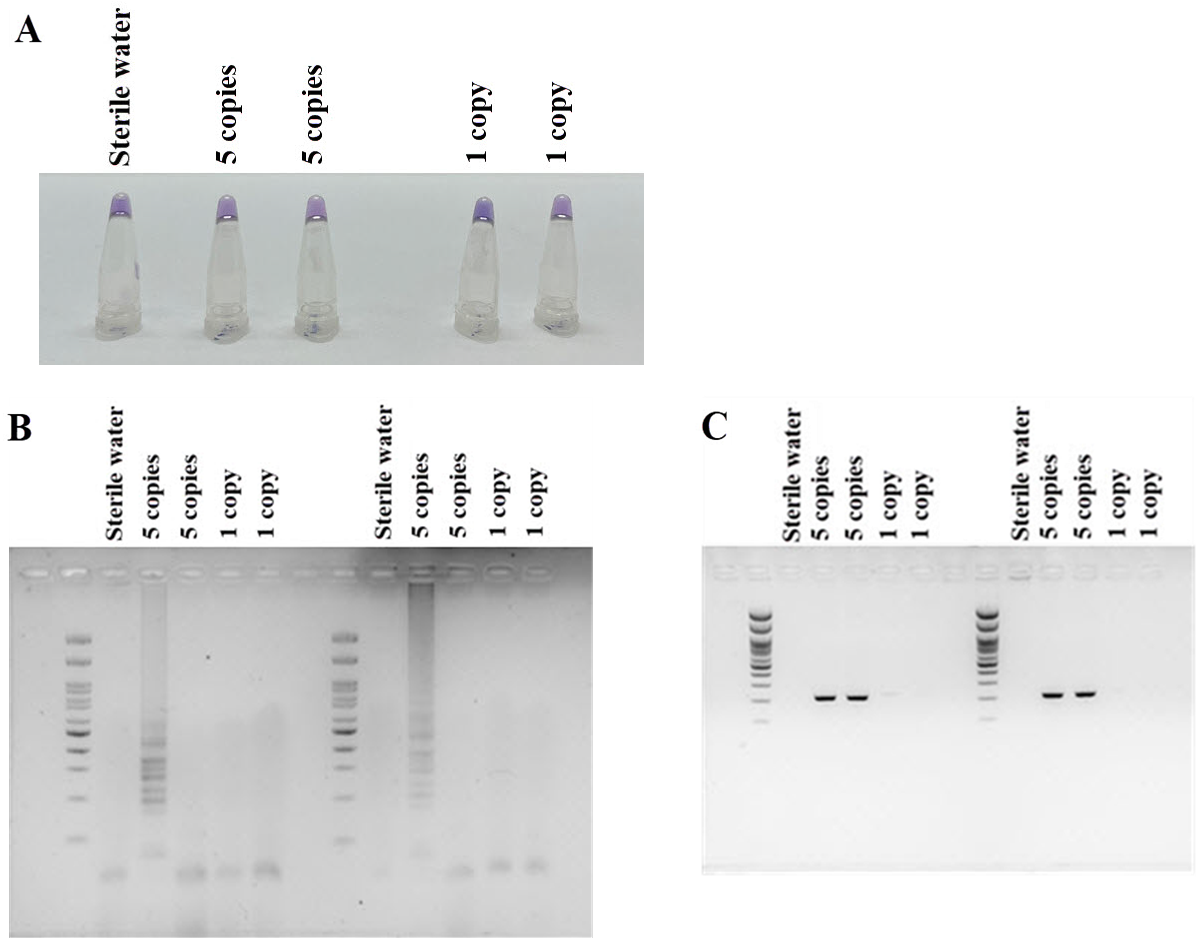

Supplement: S3 Fig — Limit of detection determination at 5 and 1 copy numbers of C. Liberibacter asiaticus omp and sterile water (negative control) by LAMP-HNB (A), LAMP-GE (B), and PCR-GE (C) assays. In (B)and (C), in addition to replicate tests of each assay in 5 and 1 copies templates, the left and right referred to two GE repeats. (TIF) [file pone.0276740.s003.tif]

Fig 1D.

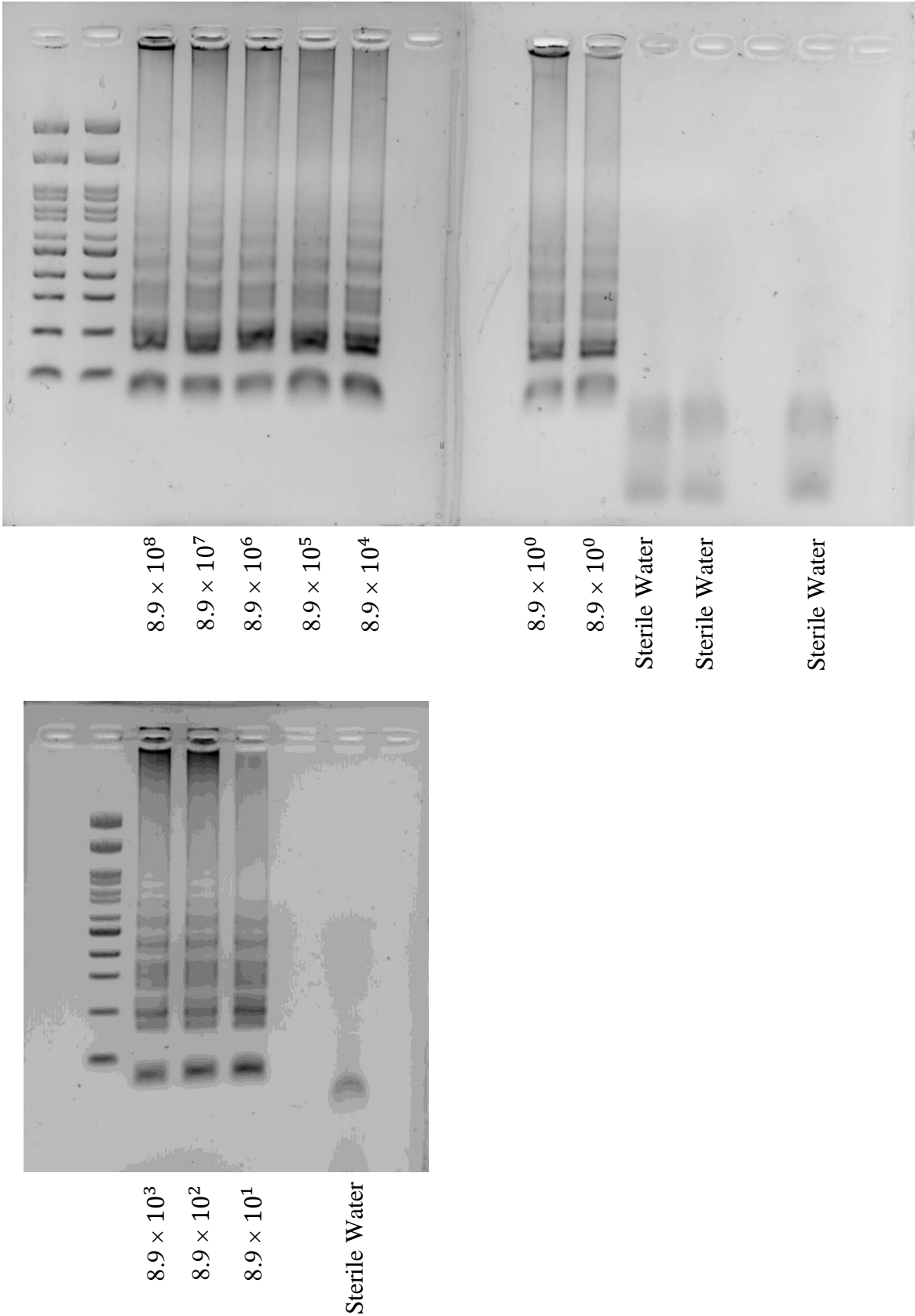

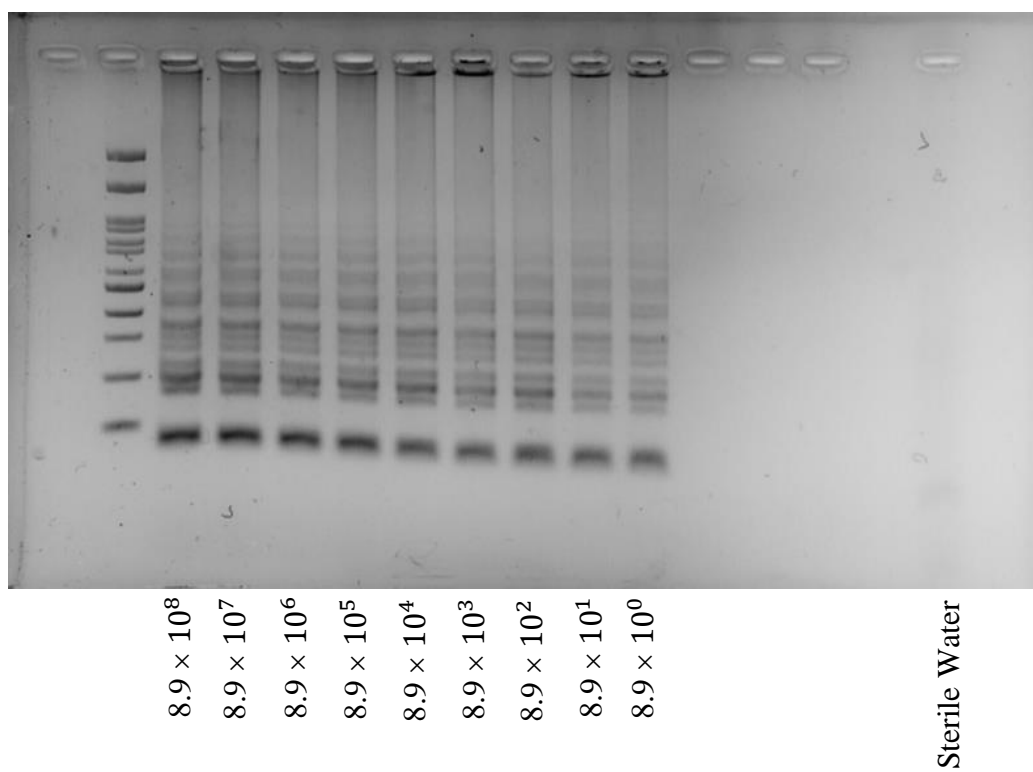

**S1 Fig.**

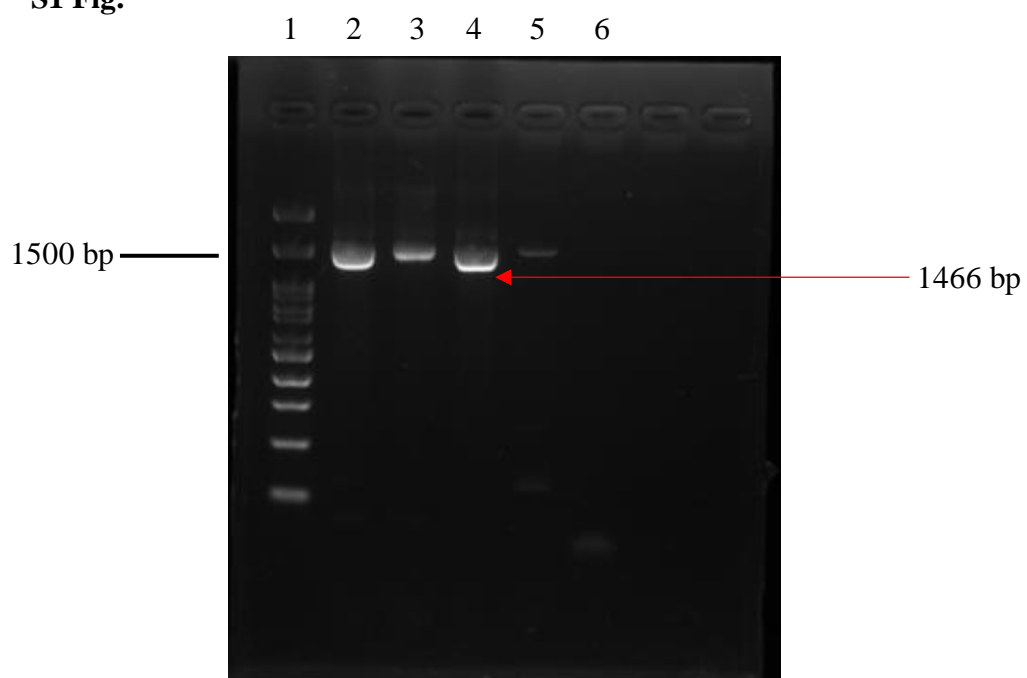

Supplement: S1 File — The other figures are of original images. (PDF) [file pone.0276740.s005.pdf]

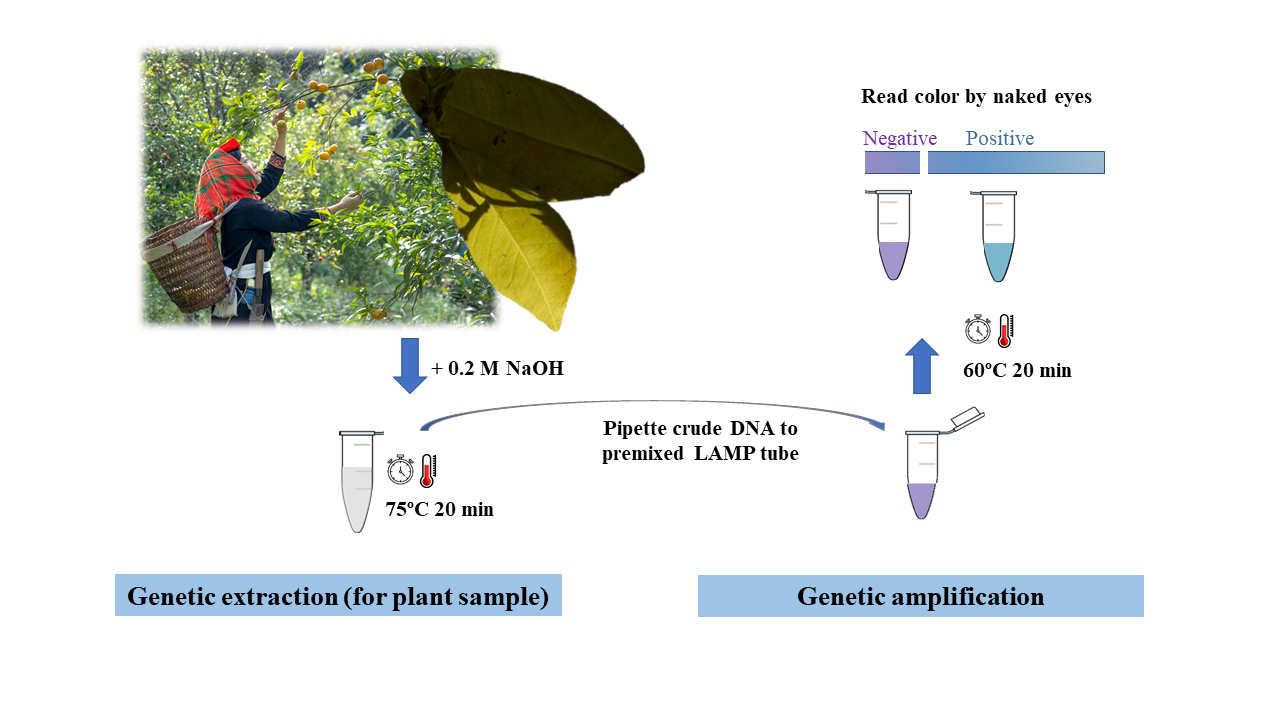

Supplement: S1 Graphical abstract — (TIF) [file pone.0276740.s006.tif]
