# Supplementary material for: Rapid visual Candidatus Liberibacter asiaticus detection (citrus greening disease) using simple alkaline heat DNA lysis followed by loop-mediated isothermal amplification coupled hydroxynaphthol blue (AL-LAMP-HNB) for potential local use
Source: PLoS One. 2022 Oct 25;17(10):e0276740. doi: 10.1371/journal.pone.0276740 (PMC9595546; doi:10.1371/journal.pone.0276740)
Supplement: S1 Table — Average extracted DNA (ng) per gram from C. reticulata leaf, bud, branch, and fruit samples (A), and BLASTN of 16S rDNA query sequence against non-redundant GenBank database for identification of bacterial pathogen (B). (PDF) [file pone.0276740.s004.pdf]

A

Sample (0.2 g)	Extracted DNA concentration in elution buffer (ng/µL)				Average extracted DNA per g plant sample weight (ng/g)
	Repeat 1	Repeat 2	Repeat 3	Avg.±S.D.	
Leaf	51.3	53.0	53.0	52.4±1.0	20,960
Bud	55.3	57.0	57.1	56.5±1.0	22,600
Branch	33.2	34.4	32.2	33.3±1.1	13,320
Fruit	21.5	20.5	24.1	22.0±1.9	8,800

B

Description	Max Score	Total Score	Query Cover	E value	Per. Ident	Accession
Candidatus Liberibacter asiaticus strain AHCA1 chromosome	562	1636	80%	4e-160	78.74%	CP029348.1
Candidatus Liberibacter asiaticus isolate HLB-M2 16S ribosomal RNA gene, partial sequence	556	556	80%	2e-158	78.58%	KY008940.1
Candidatus Liberibacter asiaticus str. gxpsy, complete genome	556	1648	80%	2e-158	78.68%	CP004005.1
Candidatus Liberibacter asiaticus haplotype H1Y 16S ribosomal RNA gene, partial sequence	556	556	80%	2e-158	78.58%	JQ867410.1
Candidatus Liberibacter asiaticus isolate 37 16S ribosomal RNA gene, partial sequence	556	556	80%	2e-158	78.58%	JN245981.1
Candidatus Liberibacter asiaticus isolate 371 16S ribosomal RNA gene, partial sequence	556	556	80%	2e-158	78.58%	GQ502291.1
Candidatus Liberibacter asiaticus strain GuangXi-GL-10-CHN 16S ribosomal RNA gene, partial sequence	556	556	80%	2e-158	78.56%	EU921615.1
Candidatus Liberibacter asiaticus 16S ribosomal RNA gene, partial sequence; 16S-23S ribosomal RNA intergenic spacer, complete sequence; and 23S ribosomal RNA gene, partial sequence	556	556	80%	2e-158	78.56%	FJ196314.1
Candidatus Liberibacter asiaticus isolate AsMo-ii 16S ribosomal RNA gene, partial sequence	553	553	80%	3e-157	78.49%	KT164832.1
Candidatus Liberibacter asiaticus strain TNal-6 16S ribosomal RNA gene, partial sequence	553	553	80%	3e-157	78.49%	KC800955.1
